# Global perspectives on lupus nephritis: a review of clinical trials and therapeutic innovations

**DOI:** 10.3389/fimmu.2026.1762892

**Published:** 2026-05-18

**Authors:** Jiaruo Xu, Yongkang Zhang, Hui Huang, Yemin Cao

**Affiliations:** 1 Innovation Research Institute of Traditional Chinese Medicine, Shanghai University of Traditional Chinese Medicine, Shanghai, China; 2 Diagnosis & Treatment Center of Vascular Disease, Shanghai TCM-Integrated Hospital, Shanghai University of Traditional Chinese Medicine, Shanghai, China; 3 Laboratory Animal Center of Traditional Chinese Medicine, Shanghai University of Traditional Chinese Medicine, Shanghai, China

**Keywords:** Clinical trials, global perspectives, lupus nephritis (LN), systemic lupus erythematosus, therapeutic innovations

## Abstract

**Background and objectives:**

Lupus nephritis (LN) remains a leading cause of morbidity and mortality in systemic lupus erythematosus (SLE). Although recent years have witnessed the approval of several targeted therapies, achieving long-term, drug-free remission remains challenging. This commentary evaluates the global landscape of interventional LN clinical trials from 2001 to 2026 to identify emerging trends and strategic gaps in drug development.

**Methods:**

We analyzed 200 interventional pharmacological trials retrieved from three major global registries: ClinicalTrials.gov, Chinadrugtrials.org, and ISRCTN. Trials were systematically categorized by study phase, geographic distribution, and therapeutic mechanism, with data synthesized to reflect the transition from non-specific immunosuppression to precision-targeted approaches.

**Key findings:**

Our analysis indicates an accelerating trend in Phase II/III trials, with a notable geographic shift toward the Asia-Pacific region. We identify a diversification of therapeutic targets beyond B-cell depletion (e.g., obinutuzumab) to include complement inhibitors, intracellular signaling blockers (BTK and JAK inhibitors), and novel immune-reset strategies such as CAR-T therapy. Despite these innovations, complete renal response rates in pivotal trials often plateau at 40-50%, suggesting a persistent “ceiling effect”.

**Conclusion and implications:**

To break current therapeutic plateaus, future research must prioritize: (1) integrating pharmacogenomics (e.g., CYP3A5 and TPMT genotyping) for personalized drug selection; (2) developing steroid-fast-tapering or steroid-free induction protocols to minimize toxicity; and (3) validating real-time molecular biomarkers to replace lagging clinical indicators. This data-driven perspective provides a practical framework for refining trial designs and achieving precision medicine in LN.

## 1 Introduction

**Figure 1:**
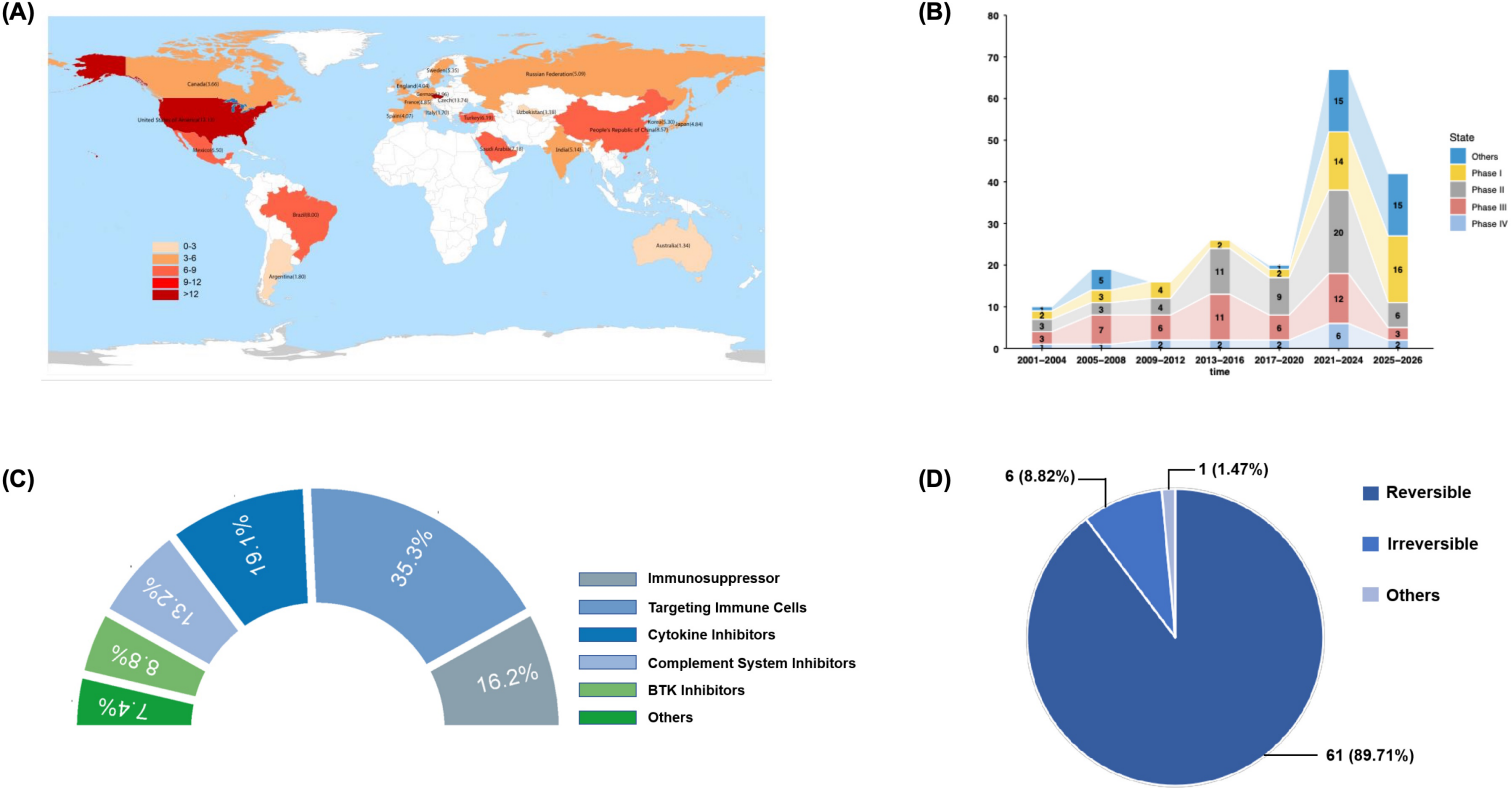
The global landscape and therapeutic evolution of clinical trials in LN (2001– 2026). **(A)** global distribution of SLE incidence. The heatmap illustrates the global burden of SLE, and the red shading indicates regions with higher reported incidence/prevalence (76, 1). **(B)** temporal trends in interventional drug trials. The bar graph depicts the number of registered interventional clinical trials for LN from January 1, 2001, to 2026. Data were aggregated from ClinicalTrials.gov, Chinadrugtrials.org, and ISRCTN. The analysis includes 200 selected trials focusing on pharmacological interventions, excluding observational studies. **(C)** classification of therapeutic mechanisms. Therapeutic agents identified in the survey (**Table 2**) are categorized by their primary mechanism of action. **(D)** reversibility of drug action. The chart illustrates the binding characteristics of the identified agents. Reversible (89.71%) refers to non-covalent binding agents (e.g., most monoclonal antibodies and reversible small molecules), whereas Irreversible (1.47%) refers to covalent inhibitors (e.g., certain BTK inhibitors) that permanently disable the target protein until new synthesis occurs. LN, lupus nephritis; SLE, systemic lupus erythematosus.

Lupus nephritis (LN) is the most common and severe renal complication associated with systemic lupus erythematosus (SLE), an autoimmune disorder characterized by widespread inflammation and tissue damage (**Figure 1A**). It affects more than 30%-60% of patients with SLE, and renal biopsy reveals evidence of renal involvement in nearly 100% of cases, underscoring the critical role of renal manifestations in the disease progression (2). Clinical manifestations of renal damage can vary widely among patients, ranging from mild proteinuria and hematuria to severe nephritis, which can lead to renal failure (3, 4). Currently, there is no standardized treatment protocol for LN. The primary goals are to control lupus activity, prevent the progression of renal damage, and minimize the side effects associated with drug therapy.

The 2025 update to the EULAR recommendations for managing LN represents a significant refinement of the therapeutic framework (5). It consolidates a more proactive and goal-oriented strategy, with complete renal remission, defined by specific thresholds of proteinuria and stable renal function, serving as the paramount treatment objective. The standard protocol consists of an induction of remission phase followed by maintenance therapy, with rigorous supportive care integrated throughout (6). For induction therapy, mycophenolate mofetil (MMF) or low-dose cyclophosphamide (CTX), each combined with glucocorticoids, remains foundational. Notably, the update elevates the role of calcineurin inhibitor-based regimens, particularly the combination of voclosporin with MMF, for high-risk or proliferative disease. Maintenance therapy continues to prioritize MMF or azathioprine, with an emphasis on adequate treatment duration. The guidance also clarifies the additive benefit of belimumab to standard therapy and suggests a broader potential role for rituximab in refractory cases. Throughout, stringent supportive care, including hydroxychloroquine use, blood pressure control, and regular monitoring via renal activity indices, is underscored as integral to management.

However, despite these advances, a significant proportion of patients fail to achieve complete renal response. This review analyzes global clinical trial data since 2000 to evaluate the evolution of treatment strategies, assess the efficacy of emerging targeted therapies, and identify critical unmet needs in LN management.

## 2 Methods

To provide a data-driven perspective on the evolution of LN therapy, we conducted a systematic search of global clinical trial registries.

### 2.1 Search strategy and data sources

We queried four major international registries: ClinicalTrials.gov, Chinadrugtrials.org, and the UK’s Clinical Study Registry (ISRCTN), among others. The search was performed for interventional trials registered from January 1, 2001, to 2026. The primary search term used was “lupus nephritis”.

### 2.2 Inclusion and exclusion criteria

Trails were included if they met the following criteria:

1. Study type: interventional drug trials;
2. Phase: trials ranging from Phase I to IV.

We excluded:

1. Observational studies and diagnostic/biomarker validation trials;
2. Non-pharmacological interventions (e.g., dietary or lifestyle-based studies);
3. Trials with a status of “Withdrawn” or “Unknown”.

### 2.3 Data extraction and categorization

A total of 200 trials were retrieved and indexed by their unique registration numbers (e.g., NCT, CTR, or ISRCTN). In this analysis, each registration number was treated as an independent entry to reflect the total volume of registered clinical activity, including multi-center extensions or platform-specific registrations. Extracted data included the trials phase, start year, intervention/treatment name, geographic region of the study sites, and LN class (see **Table 1**). To ensure scientific rigor, the therapeutic mechanism of action was cross-referenced using Pharmacodia (mdata.pharmacodia.com) and BCPM (pharma.bcpmdata.com) databases, with detailed target classifications summarized in **Table 2**.

**Table 1:** The clinical trial research worldwide from 2000 to 2026.

Registration Number	Phases	Start Time	Intervention/Treatment	Region	Types of LN
NCT00204022 (Completed)	Phase III	2001	MMF, Azathioprine	Belgium	
NCT00046774 (Completed)	Phase I	2002	MRA (anti-IL-6R monoclonal antibody)	United States	
NCT00001789 (Completed)	Phase II	2002	BG9588 (anti-CD40L Antibody)	United States	III, IV
NCT00976300 (Completed)	Phase II	2002	Cyclosporine A, Intravenous Cyclophosphamide (CTX)	Czechia	III, IV
NCT00050713 (Completed)	Phase II	2002	Sirolimus	United States	
NCT00429377 (Completed)	Phase III	2003	Tacrolimus (FK506)	Japan	
NCT01207297 (Completed)	Phase I	2003	TAC, CTX	China	III+V, IV +/- V, V
NCT00094380 (Completed)	Phase I, II	2004	CTLA4-IgG4m (RG2077)+CTX	United States	III, IV, V
NCT00089804 (Terminated)	Phase III	2004	Abetimus sodium (LJP 394)	United States, Argentina, Australia, Belarus, Brazil, Bulgaria, Czechia, Georgia, Germany, Hong Kong (China), Hungary, India, Indonesia, Italy, Lebanon, Malaysia, Mexico, Philippines, Poland, Portugal, Puerto Rico, Romania, Serbia, Slovakia, South Korea, Spain, Sri Lanka, Taiwan (China), Thailand, Ukraine	
NCT00125307 (Completed)	Phase IV	2004	TAC	Hong Kong (China)	V
NCT00268567 (Completed)	Phase II, III	2005	Leflunomide+Prednisone	China	
NCT00377637 (Completed)	Phase III	2005	MMF+CTX, MMF+Azathioprine	United States, Argentina, Australia, Belarus, Brazil, Canada, China, Czechia, France, Germany, Greece, Hungary, Italy, Mexico, Portugal, Spain, United Kingdom	
NCT00404794 (Completed)	Phase III	2005	Prednisolone and MMF, or TAC	Hong Kong (China)	III, IV
NCT00368264 (Terminated)	Phase II, III	2006	Azathioprine + Infliximab (Anti-TNF-α Chimeric Monoclonal Antibody)	Austria, Germany, Netherlands	V
NCT00282347 (Completed)	Phase III	2006	Rituximab+MMF		III, IV
NCT00371319 (Completed)	Phase IV	2006	TAC, MMF	Hong Kong (China)	III, IV, V
NCT02081183 (Terminated)	Phase III	2006	CellCept (MMF)	Venezuela	III, IV, V
NCT01845740 (Completed)	Phase I	2007	Milatuzumab	United States	
NCT00430677 (Terminated)	Phase II, III	2007	Abatacept+Corticosteroids+MMF	United States, Argentina, Australia, Belgium, Brazil, Canada, China, France, India, Mexico, Poland, Russia, South Africa, South Korea, Turkey, United Kingdom	III, IV
NCT00573157 (Terminated)	Phase II, III	2007	Atacicept+MMF+Corticosteroids	United States, Czechia, Malaysia, Singapore, Taiwan(China)	III, IV
NCT00423098 (Completed)	Phase II	2007	Mycophenolate sodium combinate with Prednisone or Methylprednisolone	Colombia, France, Germany, Greece, Hungary, Italy, Spain, Taiwan (China), United Kingdom	III, IV
NCT00425438 (Terminated)	Phase III	2007	CellCept (MMF)	China	
NCT00479622 (Terminated)	Phase I	2007	TRU-015	United States	V
NCT00709722 (Completed)	Phase I, II	2008	Deoxyspergualin (NKT-01)	Czechia, Germany	
NCT00774852 (Completed)	Phase II	2008	Abatacept+CTX	United States, Mexico	
NCT00626197 (Terminated)	Phase III	2008	Ocrelizumab		III, IV
NCT00615173 (Completed)	Phase III	2008	Tacrolimus (FK506)	China	III, IV, V
NCT00447265 (Terminated)	Phase II	2008	Etanercept	United States	
NCT00705367 (Completed)	Phase I	2008	Abatacept	China	
NCT01015456 (Terminated)	Phase III	2009	enteric-coated mycophenolate sodium (Myfortic)	Thailand	III, IV
NCT01042457 (Completed)	Phase III	2009	MMF	Thailand	III or IV (A or A/C) not include III(C) IV(C) and VI (>90% chronic irreversible scarring)
NCT00818948 (Completed)	Phase I	2009	AMG 811	United States, France, Hong Kong (China), Malaysia, Mexico	III, IV
NCT01203709 (Completed)	Phase IV	2010	low-dose combination of MMF and TAC	Hong Kong (China)	
NCT01085097 (Completed)	Phase II	2010	Laquinimod	United States, Canada, France, Russia, United Kingdom	III+/- V, IV +/- V, V
NCT01369628 (Terminated)	Phase I	2011	Atacicept+MMF	United States	III+/- V, IV +/- V, V
NCT01407406 (Completed)	Phase I	2011	BIIB023	Australia, Hong Kong (China)	
NCT01273389 (Completed)	Phase II	2011	CNTO 136	United States, Belgium, Mexico, Netherlands, Poland, Thailand	III, IV
NCT01342016 (Terminated)	Phase III	2011	TAC Capsule+Leflunomide tablet	China	III, IV
NCT01328834 (Completed)	Phase III	2011	TAC Sustained-release Capsules(ADVAGRAF)	China	III+/- V, IV +/- V, V
NCT01316133 (Terminated)	Phase IV	2011	TAC	South Korea	III, IV
NCT01284725 (Completed)	Phase III	2011	MMF, azathioprine	France	III+/-C+/- V, IV +/-C+/- V
NCT01541670 (Terminated)	Phase I	2011	Anti-Macrophage Migration Inhibitory Factor (Anti-MIF) Antibody	United States, Australia, Canada, Mexico, New Zealand	
ISRCTN84672048 (Completed)	Phase II	2011	recombinant human soluble Fc-gamma receptor IIb (SM101)	United Kingdom, Australia, Belgium, Czech Republic, France, Germany, Italy, Poland, Spain	
NCT01499355 (terminated)	Phase II	2012	BIIB023	United States, Argentina, Australia, Belgium, Brazil, Colombia, France, Germany, Hong Kong (China), Hungary, Italy, Malaysia, Mexico, Peru, Philippines, Poland, Portugal, Russia, South Korea, Spain, Thailand	III+/- V, IV +/- V
NCT01639339 (Completed)	Phase III	2012	Belimumab	United States, Argentina, Belgium, Brazil, Canada, China, Colombia, Czechia, France, Germany, Hungary, Mexico, Netherlands, Philippines, Russia, South Korea, Spain,Thailand,United Kingdom	
NCT01714817 (Terminated)	Phase III	2013	Abatacept (BMS-188667)	United States, Argentina, Australia, Brazil, Canada, Chile, China, Colombia, Czechia, India, Israel, Italy, Japan, Mexico, Peru, Puerto Rico, Romania, Russia, South Korea, Spain, Turkey	III, IV
NCT01765842 (Terminated)	Phase III	2013	Rituximab	Spain	
NCT01930890 (Terminated)	Phase II	2013	BIIB023	United States, Argentina, Australia, Belgium, Brazil, Colombia, France, Hong Kong (China), Hungary, Italy, Malaysia, Mexico, Peru, Philippines, Poland, Russia, South Korea, Spain, Thailand	
NCT01861561 (Terminated)	Phase IV	2013	CTX	Thailand	
NCT02260934 (Completed)	Phase II	2014	Rituximab+CTX/-Belimumab	United States	III+/- V, IV +/- V, V
NCT03200002 (Completed)	Phase II	2014	CTX, MMF		III, IV, V
NCT02141672 (Completed)	Phase II	2014	Voclosporin	United States, Bangladesh, Belarus, Bulgaria, China, Ecuador, Georgia, Guatemala, Mexico, Philippines, Poland, Russia, Serbia, Singapore, South Korea, Spain, Sri Lanka, Thailand, Ukraine	III, IV
CTR20140883 (Completed)	Phase III	2014	TAC, CTX	China	III+/- V, IV +/- V, V
CTR20140614 (Completed)	Phase III	2014	HE-69	China	III+/- V, IV +/- V, V
NCT02256150 (Completed)	Phase III	2014	Mizoribine (MZR)	China	III+/- V, IV +/- V, V
NCT02176486 (Terminated)	Phase I	2014	Ixazomib	United States, France, Germany, Italy, Russia, Spain, United Kingdom	III, IV, V [excluding Class III (C), IV-S (C),IVd and IV-G (C)]
NCT04318600 (Completed)	Phase I	2014	human amniotic mesenchymal stem cell		III, IV,V
NCT02547922 (Completed)	Phase II	2015	Anifrolumab	United States, Argentina, Australia, Belgium, France, Germany, Hungary, Italy, Mexico, Peru, Poland, Russia, Serbia, South Korea, Spain, Taiwan (China), United Kingdom	III+/- V, IV +/- V
NCT02550652 (Completed)	Phase II	2015	Obinutuzumab+MMF/Mycophenolic Acid (MPA)	United States, Argentina, Brazil, Colombia, Costa Rica, France, Israel, Italy, Mexico, Panama, Peru, Spain	III+/- V, IV +/- V
NCT01773616 (Terminated)	Phase III	2015	Rituximab+MMF Without Oral Steroids	United Kingdom	III, IV-S, IV-G, V
CTR20150079 (Completed)	Phase III	2015	Belimumab	China, Russia, Canada, Hungary, Colombia, Mexico, Brazil, Germany, Chile, Belgium, France, Thailand, United States, United Kingdom, Philippines, Spain, Argentina, South Korea	III+/- V, IV +/- V [excluding III(C), IV-S(C), and IV-G(C)], V
CTR20140843	Phase III	2015	BMS-188667	China, Argentina, Australia, Brazil, Canada, Chile, Colombia, India, Israel, Italy, Japan, South Korea, Mexico, Puerto Rico, Romania, Russia, Turkey, United States	III, IV [excluding III (C), IV-S (C), and IV-G (C)]
NCT02457221 (Completed)	Phase III	2015	TAC Capsules+CTX Injection	China	III+/- V, IV +/- V, V
NCT02630628 (Completed)	Phase III	2015	TAC	Hong Kong (China)	III+/- V, IV +/- V
NCT02949349 (Completed)	Phase II	2015	MMF/Azathioprine formulation		
NCT02645565 (Completed)	Phase IV	2015	CTX, Azathioprine, Methylprednisolone	India	III, IV
NCT02949973 (Completed)	Phase II	2015	Voclosporin	Malaysia	III+/- V, IV-S or IV-G, (A) or (A/C), IV +/- V, V
NCT02770170 (Completed)	Phase II	2016	BI 655064	United States, Australia, Canada, Czechia, France, Germany, Greece, Hong Kong (China), Italy, Japan, Malaysia, Mexico, Philippines, Poland, Portugal, Serbia, South Korea, Spain, Thailand, United Kingdom	III+/- V, IV +/- V
NCT02514967 (Terminated)	Phase III	2016	Blisibimod	Georgia	
NCT02682407 (Terminated)	Phase II	2016	Narsoplimab (OMS721)	United States, Hong Kong (China)	
ISRCTN47873003 (Completed)	Phase II	2016	Belimumab After B cell depletion therapy	United Kingdom	
NCT03174587 (Completed)	Phase I	2017	CS20AT04 Inj (allogenic bone marrow-derived mesenchymal stem cells)	South Korea	III, IV, V
NCT03021499 (Completed)	Phase III	2017	Voclosporin	United States, Argentina, Belarus, Brazil, Bulgaria, Canada, Chile, Colombia, Costa Rica, Croatia, Dominican Republic, Guatemala, Japan, Malaysia, Mexico, Netherlands, North Macedonia, Peru, Philippines, Poland, Puerto Rico, Russia, Serbia, South Africa, South Korea, Spain, Taiwan (China), Thailand, Turkey, Ukraine, Vietnam	III+/- V, IV +/- V, V
NCT03393013 (Completed)	Phase I, II	2018	KZR-616	United States, Australia, Colombia, Mexico, Peru, Poland, Russia, Ukraine	III+/- V, IV +/- V, V
NCT03747159	Phase III	2018	combination B-cell targeting by starting treatment with Belimumab (anti-BAFF), followed by Rituximab (anti-CD20)	Netherlands	
NCT03597464 (Completed)	Phase III	2018	MMF+Voclosporin	United States	
NCT03610516 (Completed)	Phase II	2018	CFZ533 (anti-CD40 monoclonal antibody)	Argentina, China, Germany, Hungary, Russia, South Korea, Tunisia, Turkey	III+/- V, IV +/- V
NCT04146220 (Completed)	Phase IV	2018	Prednisolone	Bangladesh	III, IV
NCT03385564 (Completed)	Phase II	2018	BI 655064	United States, Australia, Canada, Czechia, Germany, Greece, Hong Kong (China), Japan, Malaysia, Mexico, Philippines, Poland, Portugal, South Korea, Thailand, United Kingdom	III, IV
NCT03453619 (Completed)	Phase II	2018	APL-2	United States	III, IV,V
NCT04128579 (Completed)	Phase I	2019	Itolizumab [Bmab 600]	United States, India, Poland	III+/- V, IV +/- V
CTR20191068 (Completed)	Phase II	2019	CFZ533	China, South Korea, Germany, Russia, Argentina, Germany, Hungary, Russia, Tunisia, Turkey	III, IV
NCT03920059 (Terminated)	Phase IV	2019	MMF	Thailand	III or IV [exclude III(c), IV-S(c) and IV-G(c)]+/- V
NCT03943147 (Terminated)	Phase II	2019	BMS-986165	United States, Australia, Belgium, Canada, China, Czechia, Germany, Israel, Italy, Mexico, Netherlands, Russia, South Korea, Spain, United Kingdom	III, IV-S, or IV-G; or V
CTR20202662 (Completed)	Phase II	2020	Zanubrutinib Capsules	China	III+/- V, IV +/- V
CTR20200316 (Terminated)	Phase II	2020	BMS-986165	China, Argentina, Australia, Belgium, Brazil, Czech, Germany, Israel, Italy, Mexico, Holland, Russia, United Kingdom,	III+/- V, IV +/- V, V
NCT04181762 (Terminated)	Phase III	2020	Secukinumab	United States, Argentina, Australia, Brazil, Canada, Chile, China, Colombia, Croatia, Czech, Denmark, France, Germany, Greece, Guatemala, India, Italy, Japan, Mexico, Norway, Peru, Philippines, Portugal, Romania, Russia, Slovakia, South Korea, Spain, Sweden, Switzerland, Thailand, Turkey, Vietnam	III+/- V, IV +/- V, V
CTR20201175 (Terminated)	Phase III	2020	Secukinumab Injection		III+/- V, IV +/- V, V
NCT04643470 (Completed)	Phase II	2020	Zanubrutinib	China	[III(A), III (A+C), IV (A), and IV (A+C)]+/- V
NCT04376827 (Terminated)	Phase II	2020	Guselkumab	United States, Argentina, Mexico, Poland, Russia, Spain, Taiwan (China), Thailand, Ukraine	III+/- V, IV +/- V
NCT04221477	Phase III	2020	Obinutuzumab	United States, Argentina, Brazil, Colombia, France, Germany, Israel, Italy, Mexico, Peru, Poland, Russia, South Africa, Spain, United Kingdom, South Korea, Spain, United States	III+/- V, IV +/- V
ISRCTN12809537	Phase I	2021	Crovalimab	Argentina, Colombia, Germany, Italy, Spain, United States	III+/- V, IV +/- V
CTR20211541	Phase I, II	2021	MIL62	China	
NCT04868838	Phase II	2021	Daratumumab	United States	III+/- V, IV +/- V
CTR20211337	Phase II	2021	Vunakizumab (SHR-1314)	China	III+/- V, IV +/- V
NCT04702256	Phase III	2021	Oral Corticosteroids+MMF, Obinutuzumab+MMF	France	III+/- V, IV +/- V
NCT07044115	Phase I, II	2021	Recombinant Humanized Monoclonal Antibody MIL62 Injection	China	
NCT04424602 (Completed)	Phase IV	2021	CTX, Mycophenolate	Egypt	I, V, VI
NCT04564339 (Terminated)	Phase II	2021	Ravulizumab	United States, Australia, Canada, France, Germany, Italy, Netherlands, Poland, South Korea, Spain, Taiwan (China), United Kingdom	III, IV
ISRCTN80103507	Phase II	2021	ORBCEL-C™	United Kingdom	III, IV, V
CTR20222118 (Completed)	Phase I	2022	HRS-5965 tablets	China	
CTR20220218 (Completed)	Phase I	2022	MMF Capsules	China	
NCT05201469	Phase II	2022	VIB4920+MMF+ Prednisone	United States	III+/- V, IV +/- V, V
NCT03673748	Phase II	2022	Mesenchymal stem cells	Spain	III+/- V, IV +/- V
NCT05268289	Phase II	2022	Iptacopan (LNP023)	United States, Argentina, Brazil, China, Colombia, France, Germany, Hungary, India, Israel, Malaysia, Mexico, Philippines, Portugal, Puerto Rico, Singapore, Spain, Turkey, United Kingdom	III+/- V, IV +/- V
CTR20223334	Phase II	2022	Telitacicept for Injection	China	III+/- V, IV +/- V, V
NCT05126277	Phase III	2022	Ianalumab	United States, Argentina, Brazil, Canada, Chile, China, Czechia, Estonia, France, Germany, Guatemala, Hungary, India, Italy, Lithuania, Malaysia, Mexico, Romania, Singapore, South Korea, Spain, Thailand, United Kingdom, Vietnam	III+/- V, IV +/- V, V
NCT00035308 (Completed)	Phase III	2022	Abetimus sodium (LJP 394)	United States, Austria, Canada, France, Germany, Italy, Mexico, Spain, Sweden, United Kingdom	
CTR20223330 (Terminated)	Phase III	2022	Secukinumab Injection	China, Czechia, Japan, Thailand, Spain, Slovakia, South Korea, Portugal, Guatemala, Colombia, Philippines, Brazil, Vietnam	
CTR20221549	Phase III	2022	Anifrolumab Solution for Injection	China, Argentina, France, Germany, Hungary, India, Italy, Japan, Norway, Mexico, Poland, Russia, Thailand, Turkey, United States, Vietnam, Argentina, Belgium, Brazil, Bulgaria, Colombia, Malaysia, Peru	III+/- V, IV +/- V
CTR20220030	Phase III	2022	Gazyva Injection	China	III, IV, V
NCT05704088 (Completed)	Phase IV	2022	Dapagliflozin	Egypt	
NCT05232864 (Terminated)	Phase III	2022	Secukinumab	Australia, Brazil, Colombia, Czechia, Guatemala, Japan, Philippines, Portugal, Slovakia, South Korea, Spain, Thailand, Vietnam	
NCT05207358	Phase IV	2022	Rituximab, MMF, CTX, Corticosteroids	Romania	III, IV +/- V
NCT05138133	Phase III	2022	Anifrolumab	United States, Argentina, Belgium, Brazil, Bulgaria, China, Colombia, France, Germany, Hungary, India, Italy, Japan, Malaysia, Mexico, Netherlands, Peru, Poland, Puerto Rico, Russia, Thailand, Turkey, Vietnam	III+/- V, IV +/- V
NCT05780515 (Completed)	Phase I	2022	ANX009	Philippines, Taiwan (China)	III+/- V, IV +/- V
NCT05039619	Phase II	2022	Obinutuzumab	United States, Brazil, Canada, France, Italy, Mexico, Peru, Poland, Russia, South Africa, Spain, United Kingdom	III, IV [exclude isolated Class V]
NCT06342960	Phase I, II	2022	KYV-101 (anti-CD19 CAR-T cell therapy)	Germany	III, IV
NCT05748925 (Completed)	Phase IV	2022	sodium glucose linked transoprter inhibitors(SGT2i)	Egypt	
NCT05097989 (Terminated)	Phase II	2022	ALXN2050	United States, Argentina, Australia, Brazil, China, Germany, Israel, Italy, Mexico, Serbia, South Korea, Spain, Thailand, Turkey, United Kingdom	III, IV
NCT05314231 (Completed)	Phase I	2022	ALXN1720	South Korea	
CTR20234139 (Completed)	Phase I	2023	HSK39297 tablets	China	
NCT06058078	Phase II	2023	RY_SW01 Cell Injection (allogonic umbilical cord-derived mesenchymal stem cells, UCMSCs)	China	III+/- V, IV +/- V, V
NCT06155604	Phase II	2023	Dapagliflozin	Hong Kong (China)	III, IV, V
CTR20232195 (Terminated)	Phase II	2023	ALXN2050	China, United States, Mexico, Germany, Israel, Italy, Spain, Thailand, United Kingdom, Australia, South Korea, Argentina, Brazil, Turkey, Peru, Serbia	III+/- V, IV +/- V, V
CTR20230354	Phase II	2023	GR1501 injection	China	III+/- V, IV +/- V
NCT05916781	Phase IV	2023	MMF+TAC	China	
NCT05714670 (Completed)	Phase II	2023	Curcumin Oral Capsule	Egypt	
NCT05680480	Phase II	2023	Telitacicept	China	III, IV, V
NCT06406205	Phase III	2023	Voclosporin(QL1074)	China	III, IV, V
NCT06064929	Phase I	2023	Felzartamab	United States, Argentina, Australia, Canada	III, IV, V
NCT05810948 (Completed)	Phase II	2023	Efgartigimod IV	China	IV-S(C), IV-G(C), V
NCT05781750 (Terminated)	Phase II	2023	Zetomipzomib(KZR-616)	United States, Argentina, Brazil, China, Colombia, Greece, Guatemala, India, Malaysia, Philippines, South Africa, South Korea	III+/- V, IV +/- V
NCT05288855 (Terminated)	Phase III	2023	Voclosporin	United States, Colombia, Japan, Mexico, Thailand	
NCT05798117	Phase I, II	2023	CYTB323G12101	Australia, France, Germany, Spain, Switzerland	
NCT05938725	Phase I, II	2023	KYV-101 (anti-CD19 CAR-T cell therapy)	United States	III, IV
NCT05508009	Phase I, II	2023	Hematopoietic Stem Cell Transplantation (HSCT)	United States	
NCT05732402	Phase I, II	2023	Povetacicept	United States, Australia, Puerto Rico, South Korea	III+/- V, IV +/- V
ISRCTN85816718	Phase III	2023	Obinutuzumab	China	III, IV
CTR20243214 (Completed)	Phase I	2024	[14C] HSK39297	China	
NCT06350110	Phase I, II	2024	BH002 injection (Fourth-gen CAR T Cells )	China	
NCT05538208	Phase II	2024	MMF (PLUMM)	United States	III+/- V, IV +/- V
NCT06681337(Not yet recruiting)	Early Phase I	2024	BCMA CART+CD19 CART		III, IV, V
NCT07225387	Phase IV	2024	Belimumab+Voclosporin	United States	III+/- V, IV +/- V
NCT05962788 (Terminated)	Phase III	2024	Voclosporin	United States, Colombia, Japan, Mexico, Thailand	
NCT06581198	Phase II	2024	Rapcabtagene autoleucel (administered once following lymphodepletion)	United States, Australia, Austria, Brazil, Czechia, Denmark, France, Germany, Hungary, Israel, Italy, Japan, Netherlands, Norway, Romania, Saudi Arabia, Singapore, South Korea, Spain, Sweden, Switzerland, Taiwan (China), United Kingdom	
NCT06121297	Phase I, II	2024	CABA-201(an investigational cell therapy)	United States, Canada, Spain	III+/- V, IV +/- V
NCT06585514	Phase I, II	2024	CD19 CAR-T cells	China	III, IVa [excluding III (C), IV-S (C) and IV-G (C)],V
NCT06497387	Early Phase I	2024	PRG-1801(CAR-T Cells)	China	
NCT06497361	Early Phase I	2024	PRG-2311(CAR-T Cells)	China	III+/- V, IV +/- V, V
NCT06265220	Phase I	2024	AB-101(an Allogeneic Cord Blood- Derived NK-Cell Therapy)	United States	III+/- V, IV +/- V
NCT06557265	Phase I, II	2024	NKX019(a CD19 Chimeric Antigen Receptor Natural Killer (CAR NK) Cell Therapy)	United States, Puerto Rico	III+/- V, IV +/- V
NCT06434363	Phase I, II	2024	Tafasitamab	United States	
NCT06518668	Phase I	2024	NKX019 (a CD19 Chimeric Antigen Receptor Natural Killer (CAR NK) Cell Therapy)	United States	III+/- V, IV +/- V
NCT06152172	Phase I	2024	KYV 101 (CAR-T therapy)	United States	
NCT06285279	Phase I	2024	FKC288 (CAR-T Cell)	China	III, IV,V
NCT06294236	Phase I	2024	SC291 (CAR-T Cell)	United States	III, IV
NCT06375993	Phase I	2024	ADI-001 (Anti-CD20 CAR-engineered Allogeneic Gamma-Delta (γδ) T Cells)	United States	III+/- V, IV +/- V
NCT07053800(Not yet recruiting)	Phase II	2025	Obecabtagene autoleucel		III, IV, V
NCT06785519	Early Phase I	2025	CD19/BCMA Targeted CAR T-cells injection	China	III, IV, V
NCT06711887	Phase III	2025	Ianalumab	Brazil, China, Colombia, Hungary, Mexico, Romania, Singapore, South Korea, Taiwan (China), Thailand	
NCT06904729	Phase III	2025	CAR-T cells	China	III, IV
NCT06717815	Phase I, II	2025	IPG11406	China	III, IV, V
NCT06975787	Phase I	2025	Vonsetamig, Odronextamab	United States, Germany, South Korea, Spain, Taiwan (China)	
NCT07363460	Phase II	2025	HSK39297	China	III+/- V, IV +/- V
NCT07229742	Phase II	2025	SHR-2173 Injection	China	III+/- V, IV +/- V
NCT07015983	Phase II	2025	CC-97540,Fludarabine,CTX	United States, Argentina, Austria, Belgium, Brazil, Canada, Denmark, France, Germany, Israel, Italy, Japan, Netherlands, Poland, Portugal, Spain, United Kingdom	
NCT07340463	Phase II	2025	Belimumab, Telitacicept	China	III+/- V, IV +/- V
NCT06676631	Phase I	2025	NK010 or NK042 Cell Injection+Rituximab	China	III+/- V, IV +/- V
NCT06544330	Phase I	2025	SYNCAR-001 + STK-009(receptor-ligand cell therapy)	United States	III[C], IV-S[C], or IV-G[C]
NCT07038382	Phase II	2025	HLX79 (Human Sialidase Fusion Protein) in Combination With Rituximab Injection (HLX01, Anti-CD20 Antibody)	China	
NCT06943937	Phase I	2025	YTS109 cell injection(STAR-T cell therapy)	China	
NCT07000292 (Not yet recruiting)	Phase I, II	2025	MSC303	China	
NCT07035834 (Not yet recruiting)	Phase IV	2025	Gliflozin		
NCT07038447	Phase I	2025	KITE-363 (CAR T-cell Therapy)	United States, Australia, Canada	III+/- V, IV +/- V
NCT06839976	Phase I, II	2025	CD19-Directed Chimeric Antigen Receptor Autologous T Cells (CART19)	United States	III+/- V, IV +/- V
NCT06897930	Phase I, II	2025	AZD0120 (CAR-T Therapy)	United States, Australia	III, IV, V
NCT06255028	Phase I	2025	CNTY-101 (CAR iNK Cell)	United States	III+/- V, IV +/- V
NCT06708845(Not yet recruiting)	Phase I	2025	zamtocabtagene autoleucel (CAR-T therapy)		
NCT06925542	Phase I	2025	CTX112 (Anti-CD19 Allogeneic CRISPR-Cas9-Engineered T Cells)	United States, Germany	
NCT06653556	Early Phase I	2025	LCAR-AIO T cells	China	
NCT07085104	Phase I	2025	ALLO-329 (CAR T Cell)	United States, Canada	
NCT06947460	PhaseI, II	2025	CD19-BCMA CAR-T cells infusion	China	III+/- V, IV +/- V[excluding type III (C), IV-S (C), and IV-G (C)]
NCT07104721	Phase I	2025	YTS109 cell injection (STAR-T cell therapy)	China	
NCT07123519	Phase I	2025	YTS109 cell injection (STAR-T cell therapy)	China	
NCT06792799	Early Phase I	2025	KN5601(CAR NK cells)	China	
NCT07305116	Phase I	2025	UCAR T-cell	China	
NCT07201129	Phase III	2026	Cenerimod	United States	III+/- V, IV +/- V, V
NCT07323524 (Not yet recruiting)	Phase IV	2026	Dapagliflozin (10Mg Tab)	United States	III+/- V, IV +/- V, V
NCT07364396 (Not yet recruiting)	Phase I, II	2026	CRC01		III+/- V, IV +/- V
NCT06935474 (Not yet recruiting)	Phase I, II	2026	C-CAR168	United States	III+/- V, IV +/- V
NCT07412210 (Not yet recruiting)	Early Phase I	2026	AXA-NK02		III+/- V, IV +/- V
NCT07107659	Early Phase I	2026	ONT01	United States	III+/- V, IV +/- V, V
NCT07328581 (Not yet recruiting)	Phase I, II	2026	ICG318		III+/- V, IV +/- V, V
NCT07491900	Phase I	2026	HB2198(a Tetravalent Bispecific Anti-CD19/CD20 Antibody With Dual Fc Domains)	Australia	III+/- V, IV +/- V, V
NCT06984341	Phase I	2026	P-CD19CD20-ALLO1 Cells	United States	III+/- V, IV +/- V
NCT06947473	PhaseI, II	2026	umbilical cord blood CD19-BCMA CAR-T cells infusion	China	III+/- V, IV +/- V[excluding type III (C), IV-S (C), and IV-G (C)]
NCT07236762	Phase I	2026	YTS109 cell injection (STAR-T cell therapy)	China	III+/- V, IV +/- V
NCT07507201	Early Phase I	2026	UCAR T-cell	China	
CTR20261341	Phase I	2026	Cevostamab	China, Argentina, Australia, Brazil, France, Germany, Italy, Spain	III+/- V, IV +/- V

**Table 2:** Overview of therapeutic agents for LN.

Category	Agent name	Mechanism of action	Approval status	Notes	
Agents with Regulatory Approval for LN	Belimumab	BLyS (BAFF) inhibitor	FDA (2020/2023), EMA (2021), NMPA (2022)	The world's first biologic agent approved for the treatment of active LN for both adults (2020) and children (2023).	
	Voclosporin	Novel CNI	FDA (2021, in combination with MMF), EMA (2022)	The first oral medication approved for the treatment of active lupus nephritis.	
	Tacrolimus	CNI	PMDA (Japan) Approved, NMPA (China) Approved	In the United States (FDA), it has been approved for use in organ transplant rejection, and LN is an off-label use (i.e., beyond its approved indication). However, in Asia, it has been approved for use in LN.	
	Agent Name	Mechanism of Action	Status & Clinical Positioning		
Conventional Standard of Care/ Off-Label Use	MMF	IMPDH Inhibitor	Guideline Preferred First-Line Agent. FDA approved for organ transplantation. Used off-label for LN induction & maintenance.		
	MPS	IMPDH Inhibitor	Alternative to MMF (often better tolerated). Off-label use for LN.		
	CYC	Alkylating Agent	Guideline Preferred for Severe/Life-threatening LN. (Includes NIH high-dose or Euro-Lupus low-dose protocols). Approved for oncology/nephrotic syndrome, standard for severe LN.		
	HCQ	TLR 7/9 Antagonist	Base Therapy for ALL LN patients. FDA approved for SLE/Discoid Lupus. Recommended by all guidelines to prevent flares and reduce damage.		
	Glucocorticoids (Prednisone / Methylprednisolone)	Anti-inflammatory / Genomic effects	Cornerstone of Induction. Used in combination with MMF/CYC. (Pulse IV therapy followed by oral tapering).		
	AZA	Purine Synthesis Inhibitor	Mainly for Maintenance / Pregnancy. FDA approved for renal transplantation/RA. Second-line maintenance option or for pregnant patients.		
	RTX	Anti-CD20 Monoclonal Antibody	Rescue Therapy for Refractory LN. FDA approved for NHL/RA/GPA. Used off-label for patients unresponsive to MMF/CYC (based on guidelines despite LUNAR trial failure).		
	CsA	CNI	Second-line / Alternative. FDA approved for transplantation. Used in LN (especially for proteinuria reduction or pregnancy) but largely replaced by Tacrolimus/Voclosporin.		
	Leflunomide	Pyrimidine Synthesis Inhibitor	Alternative Maintenance Agent. FDA approved for RA. Used off-label for LN maintenance (especially in Asian populations/Chinese guidelines).		
	Mizoribine	IMPDH Inhibitor	Regional Use (Japan/China). Approved in Japan for LN. Rarely used in Western countries.		
	Dapagliflozin	SGLT2 inhibitor (Blocks glucose reabsorption; hemodynamic modulation)	Guideline-Recommended Adjunctive Therapy. Approved for CKD/Heart Failure. KDIGO 2024 recommends use in LN to reduce proteinuria and delay CKD progression (Non-immunosuppressive).		
	Sirolimus	mTOR inhibitor (Inhibits T-cell activation and proliferation)	Off-Label / Second-Line Option. FDA approved for renal transplantation. Used in LN for specific refractory cases or to minimize CNI/steroid toxicity (mTOR pathway blockade).		
	Agent Name	Target/Mechanism of Action	Clinical Trial Status for LN	Other Approved Indications	
Investigational Agents in Advanced Development (Phase III)	Obinutuzumab	CD20 (Type II mAb)	Phase III (REGENCY Trial). Recently met primary endpoints (2024 data)	FDA Approved for CLL / Follicular Lymphoma.	
	Anifrolumab	IFNAR	Phase III (IRIS/TULIP-LN) ongoing.	FDA Approved for SLE (non-renal) in 2021.	
	Telitacicept	Dual BLyS/APRIL inhibitor	Phase III (Global & China).	NMPA (China) Approved for SLE (2021). FDA Fast Track for SLE.	
	Dapirolizumab pegol	CD40L antagonist	Phase III (PHOENYCS GO) ongoing.	None (Investigational).	
	Ianalumab (VAY736)	BAFF-R (Depletion & Blockade)	Phase III (SIRIUS-LN) ongoing.	Investigational (Sjögren's, etc.).	
	Secukinumab	IL-17A inhibitor	Phase III (SELUNE) ongoing.	FDA Approved for Psoriasis / AS.	
	Zanubrutinib	BTK inhibitor	Phase II/III (Ongoing).	FDA Approved for Mantle Cell Lymphoma / CLL.	
	Orelabrutinib	BTK inhibitor	Phase II/III (Ongoing).	NMPA Approved for Lymphoma.	
	Iptacopan (LNP023)	Factor B (Alternative Pathway Complement Inhibitor)	Phase III (APPEAL Study) ongoing.	FDA Approved for IgA Nephropathy & PNH (Fabhalta).	
	Therapies	Agent Name	Target/Mechanism of Action	Clinical Status	Significance / Notes
Emerging Candidates in Early Phase (Phase I/II) & Novel Modalities	Cellular Therapies	CD19 CAR-T (e.g., Tisagenlecleucel, KYV-101/CABA-201)	CD19 (Chimeric Antigen Receptor T-Cell)	Phase I/II (Multiple trials ongoing).	Aiming for "Drug-free Remission" by deep B-cell depletion. High attention due to recent NEJM/Nat Med case series.
		BCMA-CD19 CAR-T	Dual CD19+BCMA	Phase I (Investigational).	Targets both B cells and Plasma cells to prevent autoantibody recurrence.
		MSCs	Immunomodulation / Tissue Repair	Phase I/II (Multiple sources: Bone Marrow, Umbilical Cord).	Focus on refractory LN. Potential to promote renal repair (unlike immunosuppressants).
	Plasma Cell Depletion	Daratumumab	CD38 (Cytolytic mAb)	Phase II / Rescue Therapy.	Approved for Multiple Myeloma. Used off-label for severe refractory LN.
		Felzartamab	CD38 (Depleting mAb)	Phase II (IGNAZ trial).	Showing promise in IgA Nephropathy, now testing in LN.
		Zetomipzomib (KZR-616)	Immunoproteasome Inhibitor	Phase Ib/II (MISSION Study completed).	Selective inhibition reduces toxicity compared to Bortezomib. Targets cytokine production and plasma cells.
		Ixazomib	20S Proteasome (Reversible inhibitor)	Phase Ib/II (Completed)	Oral proteasome inhibitor. Second-generation agent with a better safety profile than bortezomib; targets plasma cells.
	Novel Complement Inhibitors	Ravulizumab	C5 (Long-acting mAb)	Phase II (SANCTUARY).	Approved for PNH/aHUS. Investigating efficacy in TMA associated LN.
		Crovalimab	C5 (Recycling mAb)	Phase I/II (Participated in China trials).	Subcutaneous administration (Smart antibody).
		Narsoplimab	MASP-2 (Lectin Pathway)	Phase II (Investigational).	Targets the lectin pathway specifically, sparing classical/alternative pathways.
	Novel Small Molecules & Biologics	Nipocalimab / Rozanolixizumab	FcRn	Phase II (Ongoing in SLE/LN).	Accelerates IgG degradation to lower pathogenic autoantibody levels. (Novel "Medical Plasmapheresis" concept).
		Deucravacitinib	TYK2 (Select JAK family)	Phase II (PAISLEY - SLE).	More selective than Baricitinib; testing if it works for renal involvement.
		Itolizumab	CD6 (T-cell co-stimulation)	Phase I/II (EQUALISE).	Targets the ALCAM-CD6 pathway; distinct from Abatacept.
		Litifilimab (BIIB059)	BDCA2 (Dendritic Cell)	Phase II (LILAC - SLE/CLE).	Inhibits Type I IFN production at the source (pDCs). Potential for LN being explored.
		Fenebrutinib	BTK (Non-covalent inhibitor)	Phase II (Evaluated in SLE/LN)	Highly selective reversible inhibitor; aims to suppress B-cell and myeloid cell activation.
		Evobrutinib	BTK (Covalent inhibitor)	Phase II (Evaluated in SLE)	Covalent irreversible inhibitor demonstrating sustained BTK occupancy; testing efficacy in preventing flares.
		Guselkumab	IL-23p19 (Specific IL-23 antagonist)	Phase III (SELUNE Trial ongoing)	argets the IL-23/Th17 axis specifically (sparing IL-12/Th1). Key trial for measuring renal outcomes in active LN.
		Ustekinumab	IL-12/23 p40 (Dual cytokine antagonist)	Phase II Completed	Targets both Th1 and Th17 pathways. Showed promise in Phase II SLE data; paved the way for more specific IL-23 inhibitors in LN.
		Efavaleukin alfa (AMG 592)	IL-2 Mutein (IL-2 receptor agonist)	Phase II (Ongoing in SLE)	Novel Modality: Designed to selectively expand regulatory T cells to restore immune tolerance without stimulating effector T cells.
	Co-stimulation Blockade	Iscalimab (CFZ533)	CD40 (Non-depleting mAb)	Phase II Completed	Blocks CD40-CD40L interaction without depleting B cells; demonstrated proteinuria reduction.
		Dazodalibep (HZN-492)	CD40 Ligand (Fusion protein)	Phase II	Second-generation CD40L antagonist lacking Fc effector function (reduced thrombotic risk).
		Rozibafusp alfa (AMG 570)	ICOSL / BAFF (Bispecific antibody)	Phase IIb (Ongoing in SLE/LN)	Dual mechanism: Simultaneously blocks T-cell co-stimulation (ICOSL) and B-cell survival (BAFF). Novel "Two-birds-with-one-stone" approach.

LN, Lupus Nephritis; CNI, Calcineurin Inhibitor; FDA, Food and Drug Administration; EMA, European Medicines Agency; PMDA, The Pharmaceuticals and Medical Devices Agency of Japan; NMPA, The National Medical Products Administration of China. MMF, Mycophenolate Mofetil; MPS, Mycophenolate Sodium; IMPDH, Inosine Monophosphate Dehydrogenase; CYC, Cyclophosphamide; HCQ, Hydroxychloroquine; TLR, Toll-Like Receptor; AZA, Azathioprine; RTX, Rituximab; NHL, Non-Hodgkin Lymphoma; RA, Rheumatoid Arthritis; GPA, Granulomatosis with Polyangiitis; CsA, Cyclosporine A; SGLT2, Sodium-Glucose Cotransporter 2; CKD, Chronic Kidney Disease; mTOR, mammalian Target Of Rapamycin; CLL, Chronic Lymphocytic Leukemia; SLE, Systemic Lupus Erythematosus; IFNAR, Type I IFN Receptor; CD40L, CD40 Ligand; PNH, Paroxysmal Nocturnal Hemoglobinuria; CAR-T, Chimeric Antigen Receptor T-cell; CABA, Chimeric Autoantibody Receptor T-cell; NEJM, The New England Journal of Medicine; Nat Med, Nature Medicine; BCMA, B-cell Maturation Antigen; MSCs, Mesenchymal Stem Cells; aHUS, atypical Hemolytic Uremic Syndrome; TMA, thrombotic microangiopathy; FcRn, Neonatal Fc Receptor; BTK, Bruton's Tyrosine Kinase; IL, Interleukin; Th, T helper cell.

## 3 The landscape of clinical trials

### 3.1 Temporal and geographic trends

The early 2000s were characterized by pivotal trials aiming to optimize cytotoxic regimens, establishing MMF and low-dose CTX as standard induction therapies (7, 8). Subsequently, the late 2000s and post-2010 era witnessed a paradigm shift towards novel mechanism-based therapies, with a surge in industry-sponsored trials focusing on biologics (e.g., B-cell and T-cell costimulation modulators) and small molecules (9–11). Geographically, while North America and Europe have historically led trial recruitment, there has been a significant shift over the past decade with increasing contributions from the Asia-Pacific region, particularly China. This diversification is crucial, given the well-documented ethnic disparities in LN severity and treatment responses (12).

### 3.2 Analysis of target populations: the “missing” classes

A critical analysis of the enrolled patient populations reveals a distinct polarization based on histological classification. Consistent with the data in **Table 1**, many interventional trials have exclusively recruited patients with active proliferative LN (Class III/IV), with or without co-existing membranous features. This focus is driven by the aggressive clinical course of proliferative nephritis, which offers quantifiable endpoints (e.g., reduction in proteinuria and active sediment) suitable for efficacy assessment.

In contrast, other histological classes remain significantly underrepresented in the clinical trial landscape.

1. Class I and II (Mesangial LN): Specific interventional trials for these classes are virtually non-existent in our dataset. This gap is likely attributable to their relatively indolent clinical course and subtle pathological features, which typically respond well to background SLE therapy without requiring aggressive renal-specific immunosuppression. However, the lack of data leaves uncertainty regarding the optimal management of low-grade proteinuria in these patients.
2. Class VI (Advanced Sclerotic LN): Similarly, patients with advanced sclerosis (Class VI) are systematically excluded. The irreversible nature of the renal damage in this group raises ethical challenges for testing novel immunosuppressants, as the therapeutic window for recovering renal function is largely closed. Consequently, management for this subgroup remains focused on renal replacement therapy rather than immunomodulation.
3. Pure Membranous LN (Class V): While often included as a subset in mixed cohorts (Class III/IV + V), trials specifically designing primary endpoints for “pure” Class V LN are rare (only 3 trials identified in our dataset focused solely on Class V). Given its distinct pathophysiology (podocyte injury vs. inflammation) and slower progression, it may require different therapeutic strategies than proliferative disease.

### 3.3 Evolution of therapeutic mechanisms

To comprehensively evaluate the evolving landscape of drug development, we used data from mdata.pharmacodia.com and pharma.bcpmdata.com to investigate both approved and novel therapeutic agents for LN (**Table 2**). The survey results reveal a clear paradigm shift from broad-spectrum immunosuppression to precision medicine. As illustrated in **Figure 1C**, the therapeutic targets have diversified significantly. While traditional protocols relied heavily on non-specific inhibition, the current pipeline focuses on specific immunological pathways, predominantly including B cells (35.3%), cytokines (19.1%), and intracellular signaling pathways (8.8%).

Notably, recent years have witnessed the rise of novel mechanisms such as complement inhibition (13.2%) and immunoproteasome modulation (16.2%), reflecting a deeper understanding of LN pathogenesis beyond simple autoimmunity. Furthermore, the mode of action is evolving; as shown in **Figure 1D**, most agents exert reversible inhibitory effects (89.71%), allowing for flexible management of immune suppression, whereas irreversible inhibitors (e.g., certain Bruton’s tyrosine kinase inhibitors) represent a smaller but potent niche. This evolution highlights a strategic pivot towards balancing high efficacy with reduced systemic toxicity.

## 4 Emerging therapeutic targets and clinical evidence

Based on the mechanistic evolution outlined above, we critically assess the clinical potential of key emerging targets, focusing on those that address the limitations of conventional broad-spectrum immunosuppression.

### 4.1 B-cell directed therapies

#### 4.1.1 CD20 depletion

Historically, the use of rituximab (a chimeric Type I anti-CD20 antibody) in LN was met with disappointment in the pivotal Phase III LUNAR trial, which failed to meet its primary endpoint of superior renal response compared to placebo (13, 14). Critics have attributed this failure not to a lack of biological efficacy, as serological improvements were observed, but to limitations in trial design, particularly the confounding effect of high-dose background corticosteroids and incomplete B-cell depletion in tissue niches (15, 16).

Addressing these shortcomings, obinutuzumab, a novel humanized Type II anti-CD20 antibody, has recently demonstrated superior efficacy (17). Unlike rituximab, obinutuzumab is glycoengineered to enhance antibody-dependent cellular cytotoxicity (ADCC) and induce direct cell death, resulting in more profound B-cell depletion (18). The Phase III REGENCY trial recently reported positive top-line results, meeting its primary endpoint of Complete Renal Response (CRR) at Week 76 (19). This success validates the hypothesis that deeper and more sustained B-cell depletion is required to translate biological activity into clinical renal remission in LN.

#### 4.1.2 B-cell survival factors (BAFF/APRIL)

While CD20 antibodies target circulating B cells, they spare long-lived plasma cells. Targeting B-cell survival factors offers a complementary approach.

The success of the BLISS-LN trial established belimumab as the first FDA-approved biologic for LN (20, 21). In the pivotal Phase III BLISS-LN trial, belimumab significantly improved the Primary Efficacy Renal Response (PERR) rate compared to placebo and reduced the risk of renal-related events or death, with a safety profile comparable to standard therapy alone. Inhibiting B-lymphocyte stimulator (BLyS), it effectively reduces the transition of naive B cells to autoreactive plasma cells (22). Its inclusion in the 2024 KDIGO guidelines as an add-on therapy underscores its role in enhancing renal response rates and sparing glucocorticoids (23).

Taking this mechanism a step further, telitacicept (RC18) targets both BLyS and a proliferation-inducing ligand (APRIL) (24). Since APRIL is crucial for the survival of long-lived plasma cells, which produce autoantibodies, telitacicept theoretically offers broader suppression of the pathogenic humoral response than BLyS inhibition alone. Clinical data from Phase IIb and recent Phase III studies in China have shown promising renal recovery rates and significant reductions in proteinuria, positioning it as a potent alternative for refractory cases (25, 26).

The evolution of B-cell therapies highlights a fundamental shift in philosophy. Early trials simply aimed to “remove” B cells, often failing because pathogenic clones persisted in protected tissue niches. The current trend is moving toward “immune reset”. Strategies like obinutuzumab aim for absolute depletion, while emerging CAR-T therapies (discussed in Section 3.4.2) seek to completely purge the immunological memory. However, the next leap forward must address B-cell repopulation: rather than continuous suppression, future trials should investigate how to guide the immune system to repopulate with a “tolerant” B-cell repertoire, potentially through the integration of pharmacogenomic markers such as FCGR3A variants to predict individual response.

### 4.2 Complement inhibition

Complement activation, evidenced by hypocomplementemia and glomerular deposition of C3/C4, is a hallmark of active LN (27). However, targeting the complement system remains a “double-edged sword”, as excessive inhibition may hinder the clearance of immune complexes and increase infection risks.

#### 4.2.1 Terminal pathway blockade (C5 inhibitors)

Agents like ravulizumab and crovalimab prevent the formation of the membrane attack complex (MAC) (C5b-9) (28–33). While effective in managing LN-associated thrombotic microangiopathy (TMA), blocking the terminal pathway alone may be “too downstream” to fully suppress C3b-mediated opsonization and inflammatory cell recruitment. Critically, the loss of MAC function introduces a persistent risk of encapsulated bacterial infections (e.g., Neisseria meningitidis), necessitating strict vaccination protocols (34). It’s a significant clinical burden for patients already on complex immunosuppressive regimens.

#### 4.2.2 Alternative pathway blockade (factor B inhibitors)

The oral Factor B inhibitor iptacopan (NCT05268289) offers a more proximal intervention by blocking Factor B and halting the amplification loop of the alternative pathway (35). This approach theoretically provides superior control over glomerular inflammation compared to terminal blockade alone (36, 37). However, the critical concern remains that proximal inhibition more broadly impairs the body’s natural “clearing” mechanism and anti-bacterial defense, potentially magnifying the infection risks seen with terminal blockade (38). Early classical pathway components (C1q, C4) are protective by facilitating the clearance of apoptotic debris (39). Current trials often struggle to selectively block the “inflammatory” pathways that damage the kidney, while sparing the “protective” pathways that help the body clear metabolic waste. Future success may hinge on temporally specific modulation rather than continuous broad inhibition.

### 4.3 Intracellular signaling

While biologic agents target cell surface receptors or circulating cytokines, small molecule inhibitors capable of penetrating the cell membrane offer a unique advantage: the ability to block downstream signaling cascades shared by multiple pathogenic receptors.

#### 4.3.1 BTK inhibition: dual targeting of B cells and myeloid cells

Bruton’s tyrosine kinase (BTK) is a critical node in B-cell receptor (BCR) signaling. However, unlike CD20 antibodies that primarily deplete B cells, BTK inhibitors (BTKi) offer a dual mechanism of action (40). They block BCR signaling in B cells and, crucially, inhibit Fc gamma receptor (FcγR) signaling in myeloid cells (macrophages and monocytes), which are key effectors of renal tissue injury (41, 42). Our dataset highlights zanubrutinib, a highly selective second-generation BTK inhibitor, which is currently under evaluation in a Phase II trial for active proliferative LN (CTR20202662). Owing to its optimized molecular structure, zanubrutinib demonstrates greater selectivity for BTK and a reduced off-target toxicity profile compared to first-generation agents such as ibrutinib (43, 44). The theoretical advantage of BTKi lies in its potential to suppress autoantibody production while simultaneously dampening the intrarenal inflammatory milieu driven by macrophages (45, 46). However, the efficacy signal in SLE/LN has been mixed in the broader class (e.g., failures of other BTKi in SLE phase II trials), suggesting that patient selection based on molecular signatures (e.g., high BTK/myeloid gene expression) may be necessary to unlock their full potential (47–49).

#### 4.3.2 JAK inhibition: broad cytokine blockade

The pathogenesis of LN involves a complex storm of cytokines (Type I Interferons, IL-6, IL-12/23) (50). Targeting single cytokines often proves insufficient. Janus kinase (JAK) inhibitors can simultaneously interrupt signaling from multiple cytokine receptors, acting as “broad-spectrum” cytokine blockers (51). As shown in **Table 1**, baricitinib (a JAK1/2 inhibitor) has advanced to Phase III clinical evaluation (e.g., NCT05432531). Early phase data suggested that baricitinib could significantly reduce proteinuria and improve resolution of renal flares (52). The oral bioavailability and rapid onset of action make JAK inhibitors attractive options. However, safety concerns, particularly regarding venous thromboembolism (VTE) and herpes zoster reactivation, require vigilant monitoring (53–55). Balancing the depth of immune suppression with infection risk remains the primary challenge for this class in the context of LN.

### 4.4 Novel approaches

Beyond established pathways, the LN pipeline is expanding into highly innovative modalities. These therapies represent high-risk, high-reward strategies targeting the fundamental machinery of autoimmunity and tissue repair.

#### 4.4.1 Immunoproteasome inhibition

The immunoproteasome is highly expressed in immune effector cells and is crucial for cytokine production and plasma cell survival (56). Unlike broad proteasome inhibitors (e.g., bortezomib), which carry significant toxicity, selective immunoproteasome inhibitors aim to target pathogenic cells while sparing constitutive proteasomes in non-immune tissues (57, 58). Our analysis identified zetomipzomib (KZR-616) as a first-in-class selective inhibitor of the immunoproteasome (NCT03393013) (59). Results from the Phase Ib/II MISSION study demonstrated a favorable safety profile and clinically meaningful reductions in proteinuria and SLEDAI scores in patients with active LN, while avoiding the severe peripheral neuropathy and systemic toxicity associated with bortezomib (35). Simultaneously targeting cytokine release and plasma cell function, it offers a dual therapeutic effect distinct from standard immunosuppressants.

#### 4.4.2 Chimeric antigen receptor-T cell therapy

The concept of “immune reset” in refractory LN is not entirely new, having been fundamentally established by Hematopoietic Stem Cell Transplantation (HSCT). For decades, HSCT has served as the most extensively studied strategy for systemic immune reconstitution in severe SLE/LN, demonstrating the potential for long-term, drug-free remission through the eradication of autoreactive immunological memory (60). However, the widespread clinical application of HSCT has been significantly hampered by its high treatment-related toxicity and the risks associated with non-selective lymphodepletion. Modern engineered cellular therapies, led by CAR-T, represent a precision evolution of this “reset” philosophy. By specifically targeting B-cell lineages (e.g., CD19 or BCMA) rather than the entire immune system, CAR-T aims to achieve the profound efficacy of HSCT while significantly mitigating systemic risks, thus marking a transition from broad-spectrum ablation to targeted immune-reconstitution (61).

Recent entries in the registry highlight the initiation of trials using CD19/BCMA bispecific CAR-T cells (e.g., NCT05030779) and fourth-generation CAR-T constructs (e.g., NCT06350110, initiated in 2024) (62). While data are preliminary, early case reports suggest that CAR-T therapy can induce drug-free remission in severe, multidrug-resistant LN. However, the risks of cytokine release syndrome (CRS) and neurotoxicity require careful patient selection and specialized management (63).

#### 4.4.3 Mesenchymal stem cell therapy

Shifting focus from suppression to repair, MSCs offer immunomodulatory and tissue-regenerative properties (64). Multiple trials in our dataset (e.g., NCT03174587) investigate allogenic MSCs derived from bone marrow or umbilical cord (65). These therapies aim to downregulate autoreactive T/B cells and promote renal tissue repair. While early phases indicate safety, consistent efficacy in large-scale randomized controlled trials (RCTs) remains to be firmly established, with standardization of cell preparation being a key hurdle.

## 5 Discussion and future perspectives

Despite the unprecedented expansion of the therapeutic armamentarium over the past two decades, a definitive “cure” for LN remains elusive. Our review of the global trial landscape reveals a paradox: while the number of targeted agents has surged, the “ceiling” of complete renal response (CRR) in pivotal Phase III trials remains stubbornly around 40-50% at one year (21, 66, 67). This stagnation suggests that simply adding more immunosuppressants to current protocols may yield diminishing returns. Based on the evidence synthesized, we propose three priority areas for future research.

Current trials largely treat active LN as a homogeneous entity, recruiting “all-comers” with proliferative histology. However, LN is biologically heterogeneous. For instance, a patient driven by a Type I Interferon signature (responsive to anifrolumab) may differ fundamentally from one driven by B-cell selection survival (responsive to belimumab/telitacicept) (68). Future trials must move beyond histological classification. We advocate for the integration of liquid biopsy (e.g., urine proteomics) and molecular stratification into trial inclusion criteria (69). For example, trials for BTK inhibitors could stratify patients based on myeloid signature expression (42). Only by matching the drug’s mechanism to the patient’s specific immunopathology can we break the 50% response barrier.

Most trials analyzed in this review employed an “add-on” design (Standard of Care + Experimental Drug vs. Standard of Care + Placebo). While ethical, this design often masks the efficacy of the novel agent due to the high potency of background high-dose corticosteroids and MMF. The failure of several biologically potent agents (e.g., ocrelizumab, abatacept) in LN may partly be attributed to this “noise”. The success of the recent voclosporin and belimumab trials, which enforced stricter steroid tapering, points the way forward. Future designs should prioritize superiority trials with rapid steroid withdrawal or even head-to-head comparisons against MMF, rather than simply piling new drugs on top of toxic backbones.

Proteinuria, the traditional primary endpoint, is a lagging indicator of renal inflammation. Persistent proteinuria can reflect chronic damage rather than active disease, potentially leading to overtreatment. Conversely, histological activity can persist despite clinical remission. There is an urgent need to validate non-invasive biomarkers (e.g., urinary CD163, ALCAM) as surrogate endpoints that can read out renal inflammation in real-time. Furthermore, as “drug-free remission” becomes a tangible goal with therapies like CAR-T, future trials must incorporate long-term follow-up (3–5 years) to assess not just response induction, but the durability of tolerance and the prevention of end-stage kidney disease (ESKD).

Beyond clinical and histological stratification, the integration of pharmacogenomics is essential for a truly precise approach to LN management. Genetic variations in drug-metabolizing enzymes and immune receptors significantly influence both therapeutic efficacy and systemic toxicity. For instance, screening for TPMT and NUDT15 polymorphisms is increasingly recognized as a clinical imperative to prevent severe bone marrow suppression in patients receiving azathioprine (70, 71). Similarly, the metabolism of calcineurin inhibitors (CNIs), such as tacrolimus and the recently approved voclosporin, is heavily dictated by CYP3A5 genotypes, where “rapid metabolizers” may fail to reach therapeutic troughs despite standard dosing (72). Furthermore, polymorphisms in the FCGR3A gene, which encodes the FcγRIIIa receptor on effector cells, may modulate the intensity of antibody-dependent cellular cytotoxicity (ADCC), potentially explaining the heterogenous clinical response to B-cell depleting agents like rituximab and obinutuzumab (73, 74). Identifying these single nucleotide polymorphisms (SNPs) before therapy initiation offers a transformative pathway to minimize trial-and-error prescribing and the “ceiling effects” currently observed in LN clinical trials.

The last 20 years of clinical trials have transformed LN from a disease managed by blunt immunosuppression to one treated with increasing precision. The approvals of belimumab and voclosporin mark the beginning of this new era. However, the next leap forward will not come from merely discovering new targets, but from revolutionizing how we select patients and design trials. By embracing precision medicine and prioritizing steroid-free remission, the next decade holds the promise of altering the natural history of this devastating disease.

## Data Availability

The original contributions presented in the study are included in the article/supplementary material. Further inquiries can be directed to the corresponding authors.
